# Nanocarrier-mediated delivery of α-mangostin for non-surgical castration of male animals

**DOI:** 10.1038/s41598-017-16563-3

**Published:** 2017-11-24

**Authors:** Jakarwan Yostawonkul, Suvimol Surassmo, Katawut Namdee, Mattaka Khongkow, Chatwalee Boonthum, Sasithon Pagseesing, Nattika Saengkrit, Uracha Rungsardthong Ruktanonchai, Kaywalee Chatdarong, Suppawiwat Ponglowhapan, Teerapong Yata

**Affiliations:** 1 National Nanotechnology Centre (NANOTEC), National Science and Technology Development Agency, Pathumthani, Thailand; 2 Department of Obstetrics, Gynaecology and Reproduction, Faculty of Veterinary Science, Chulalongkorn University, Bangkok, Thailand

## Abstract

The overpopulation of abandoned and stray companion animals has become a global crisis. The main purpose of this study was to develop a novel nanomedicine-based antifertility compound for non-surgical castration of male animals. Mangosteen (*Garcinia mangostana* L) pericarp extract has been shown to exhibit anti-fertility property. α-mangostin (AM)-loaded nanostructured lipid carrier (AM-NLC) was developed to improve male germ cell apoptosis. This study was conducted to investigate physicochemical properties of AM-NLC and determine the biological effects of AM-NLC on spermatogonia cells and testicular explants obtained from castrated testes. AM-NLC was produced through a hot homogenization technique. The negatively charged particle of AM-NLC was nano-sized with a narrow dispersity. AM-NLC exhibited antiproliferative activity towards spermatogonium cells. It induced apoptosis in the cells. In addition, AM-NLC exhibited anti-inflammatory activities in lipopolysaccharide-activated macrophages. Abnormal anatomy of seminiferous tubule was noted following treatment of testicular explant with AM-NLC. This nanomedicine-based sterilant would be a promising platform that may have utility in non-surgical castration of male animals by intra-testicular injection.

## Introduction

It is well established that the most effective means of controlling pet populations is sterilization^1^. Yet with the large numbers of owned and stray cats and dogs in resource-poor developing countries, surgical castration programmes currently available are not effective and sufficient as it requires anesthesia, suitable surgical equipment and facility, and adequate recovery time^2^.

Recent research into the contraception and sterilization for controlling the reproduction of animals has focused on non-surgical methods. Intra-testicular injection of anti-fertility compounds, more commonly known as chemical castration, have been exploited. This method requires the direct injection of chemical sterilants into the testicles that causes permanent infertility in male animals^3^. Products in development for intra-testicular injection include calcium chloride^1^ and a zinc-based solution^4,5^.

The US Food and Drug Administration (FDA) has approved Zinc Gluconate as a chemical sterilant for use in male dogs^6^. Unfortunately, zinc gluconate-based products have been reported to cause adverse effects. Excessive scrotal swelling and inflammation were noted^7^. This chemical sterilant is a cytotoxic substance that induces necrosis and inflammation of testicular cells, resulting in atrophy of the testes^8^.

Programmed cell death by apoptosis is more preferable than necrosis in eliminating unwanted cells because it does not trigger inflammation to neighboring cells^9^. Therefore, induction of apoptosis has emerged as an attractive approach for the animal sterilization^10–13^.

Nanostructured lipid carriers (NLC) are a nano-particulate carrier system in which partial-crystallized lipid particles (average diameter ≤100 nm) are dispersed in an aqueous phase containing emulsifier(s)^14^. NLC have recently proven to be advantageous over other colloidal carriers in delivering pharmaceutical drugs due to their high drug loading, encapsulation efficiency and stability. They also increase bioavailability and stability of bioactive compounds and provide controlled release of encapsulated materials^15,16^.

In order to obtain a safe, effective, affordable, and permanent non-surgical form of contraception, we have developed a new generation of nanomedicine-based sterilant for non-surgical castration of male animals. This particular nanostructured lipid carrier (NLC) encapsulated the pure natural compound α-mangostin (an apoptosis-inducing and anti-inflammatory agent) derived from mangosteen pericarp extract (*Garcinia mangostana* Linn) which is a tropical tree mainly found in South East Asia and considered to be “the queen of fruits”. This compound has been also found to possess a broad spectrum of pharmacological effects, such as anti-cancer, anti-microbial, anti-oxidant, cardioprotective, anti-diabetic, and anti-obesity activities^17,18^.

Therefore, the main purpose of this study was to investigate its physiochemical properties as well as to evaluate its potential as a chemical sterilant for non-surgical castration of male animals.

## Materials and Methods

### NLC preparation

α-mangostin-loaded NLC was prepared using hot and high pressure homogenization techniques. Lavender essential oil was used as liquid while Cetyl palmetate was solid lipid, respectively. Briefly, lavender oil (4.0 g) was weighed and warmed in a water bath at 70 °C. The desired amount of α-mangostin (0.4 g) was dissolved in warmed lipid carrier until completely dissolved while α-mangostin was not loaded for blank-NLC. Then, Cetyl palmetate (6.0 g) and Montanov 82 (2.0 g) were added into warmed lipid carier which used as oil phase. Polyoxyethylene (20) sorbitan monolaurate, poloxamer (1.0 g) and glycerol (5.0 g) were dissolved in pure water, and warmed at 70 °C in a water bath as aqueous phase. Then, aqueous phase was added into oil phase under mechanical stirring at 300 rpm for 3 min in a water bath at 70 °C as a pre-emulsion. The pre-emulsion was homogenized by using high speed homogenizer (IKA, Altra-Turrac T25, Germany) at 8000 rpm for 3 min. Then, they were homogenized by using high pressure homogenizer at 1500 bars for 3 cycles (Avestin, EmulsiFlex-C3, Germany). Throughout this paper, the abbreviation AM-NLC is used to refer to α-mangostin-encapsulated NLC.

Encapsulation efficiency (EE) of AM-NLC were determined. Briefly, AM-NLC were added onto Amicon membrane filter (Amicon Ultra-15, Merch Millipore Ltd., Darmstadt, Germany). Filled amicon tube were centrifugation at 8000 g for 1 h. Unencapsulated α-mangostin in the aqueous phase (supernatant) was filtered through a 0.22 µm nylon filter and injecting to high performance liquid chromatography (HPLC) (Waters, e2695, Singapore) with Photodiode array detection (PAD) (Waters, 2998 Photodiode Array, Singapore). EE were calculated following Equation:

$$ \% EE=\frac{{C}_{i}-{C}_{F}}{{C}_{i}}\,\times 100$$

where *C*_*i*_ represents initial concentration of α-mangostin added to NLC particles; *C*_*f*_ represents the concentration of unencapsulated α-mangostin.

### Characterization of NLC particles and their stability

Dynamic light scattering (DLS) and zeta potential of prepared blank NLC and AM-NLC were determined using a Malvern Instruments Zetasizer Nano ZX. DLS measurements were carried out using He-Ne laser (λ_0_ = 633 nm, θ = 173°). The particle solution was diluted 1000 times in pure water before measurement. All measurements were performed in triplicate at 25 °C. The particle morphology of NLC and AM-NLC were characterized using transmission electron microscope (JEM-2100 plus, JEOL, Japan) after vacuum-drying. Briefly, the sample were diluted in pure water and dropped directly on copper grid, the samples were observed with magnification of 200.0kX.

### *In vitro* release study

The experiment was performed to evaluate AM release from AM-NLC. The sample was loaded at 200 µl into Franz cell released container over 500 kDa Ultrafiltration Discs (Biomax®, Millipore, USA) for separating the nanoparticle (with entrapped drug) and free drug, which was accurately controlled at 37 °C and under PBS buffer pH 7. At fixed time intervals, sampling from the receiver solution was undertaken and directly injected into HPLC-UV. The amount of released α-mangostin was analyzed by HPLC-UV technique. For comparison, the release study of free unentrapped AM and poorly water-soluble AM were also performed under the same conditions as the AM-NLC. The relative amounts of individual components are based on peak areas obtained.

### Cell culture

The GC-1 murine spermatogonia cell line (ATCC) and the BJ foreskin fibroblast cell line (ATCC) were maintained in complete D-MEM medium supplemented with 10% Fetal Bovine Serum (FBS), Penicillin (100 units/ml), Streptomycin (100 µg/ml) and L-glutamine (2 mM). The RAW264.7 murine macrophage cell line (ATCC) was grown in RPMI 1640 supplemented with 10% FBS, 100 units/ml penicillin, 100 units/ml streptomycin and 2 mM L-glutamine. Cells were grown at 37 °C in a humid atmosphere of 5% CO_2_.

### Cell viability and apoptosis assays

Cell viability was evaluated by CellTiter-Glo® Luminescent Cell Viability assay system (Promega). Cells were seeded at a density of 5 × 10^4^ cells/well in 48-well plates and allowed to grow until 70–80% confluent followed by treatment with NLC or chemicals as indicated by the experiment. Cytotoxicity was examined 24 h post incubation. Cells were also stained with the reagents in the LIVE/DEAD® Cell Viability/Cytotoxicity Assay Kit (Invitrogen). Caspases 3/7 activity and Annexin V-FITC/propidium iodide (PI) staining as indicator of apoptosis/necrosis were examined according to the manufacturer’s instructions.

### Anti-inflammatory activity

RAW 264.7 cells were grown in 48-well plates at a density of 5 × 10^4^ cells/well for 24 hours until reaching 70–80% confluence. After incubating cells with blank NLC or AM-NLC for 30 minutes, cells were stimulated with 0.1 µg/ml LPS. Following 24-h incubation, two aliquots of cell supernatant were processed as separate samples for determination of nitrite production and quantification of tumor necrosis factor (TNF)-α by enzyme-linked immunosorbent assay (ELISA), respectively. Cell supernatant collected from cells in which were stimulated with LPS only was used as control. Detection of accumulated nitrites (NO_2_^−^) in the cell supernatants was performed using the Griess reagent (Sigma). 150 μl of the cell supernatant was incubated with 150 μl Griess reagent for 15 min in a dark at room temperature and the absorbance was measured at 546 nm on a UV/Vis microplate reader. The concentrations of nitrites were derived by regression analysis using serial dilutions of sodium nitrite as a standard. Quantification of TNF-α was performed based on the use of the ELISA assay according to the manufacturer’s instructions of the TNF-α Mouse ELISA Kit (ThermoFisher Scientific) with final measurements by using a microplate reader.

### Testes tissue explant culture and histological analysis

Castrated testes from pubertal cats (n = 3) which served as sources of donor testicular tissues were obtained from small animal hospital, Chulalongkorn University, Bangkok, Thailand, and used to establish the *Ex vivo* testicular explants. Testes from pet cats (regularly castrated at small animal hospital) are considered to be animal remains awaiting disposal. Ethical approval from the Institution is therefore not required. Pieces (10–20 mg) were removed from the testes and placed on 35 mm culture dishes (Corning) individualized by donor. Ten sections of testicular tissue from each donor were placed on a 0.45-μm pore membrane (Millipore, Bedford, MA) in a single well within a six-well culture plate. One plate was used for each donor, equaling 60 sections per donor. Explants were maintained in D-MEM culture medium containing 10% FBS, 100 units/ml penicillin, 100 units/ml streptomycin and 2 mM L-glutamine at 32 °C in a humidified atmosphere of 5% CO_2_ in air. After 24 h incubation with NLC or AM-NLC, explants were place in Glo Lysis Buffer (Promega), homogenized with PYREX® 3 mL Glass Pestle Tissue Grinder, and centrifuged at 20,000 x g for 10 min. Cell viability were determined using CellTiter-Glo® Luminescent Cell Viability assay system (Promega). The experiments were replicated using three different donor animals. For histological analysis, testicular explants were fixed in 4% paraformaldehyde in PBS overnight, dehydrated by immersion in a series of graduated alcohol concentrations, embedded in paraffin, sectioned at 5.0 μm. Sections were stained with hematoxylin to visualize cell nuclei. Slides were evaluated under inverted light microscopy.

### Statistical analysis

GraphPad Prism software (version 5.0) was used to perform statistical analyses. One-way analysis of variance, or repeated measures analysis of variance, followed by Tukey post-hoc tests were used for multiple comparisons. A value of p < 0.05 was considered statistically significant and denoted as follows: *p < 0.05, **p < 0.01 and ***p < 0.001.

## Results

### Synthesis and Physicochemical characterization of NLC

**Figure 1:**
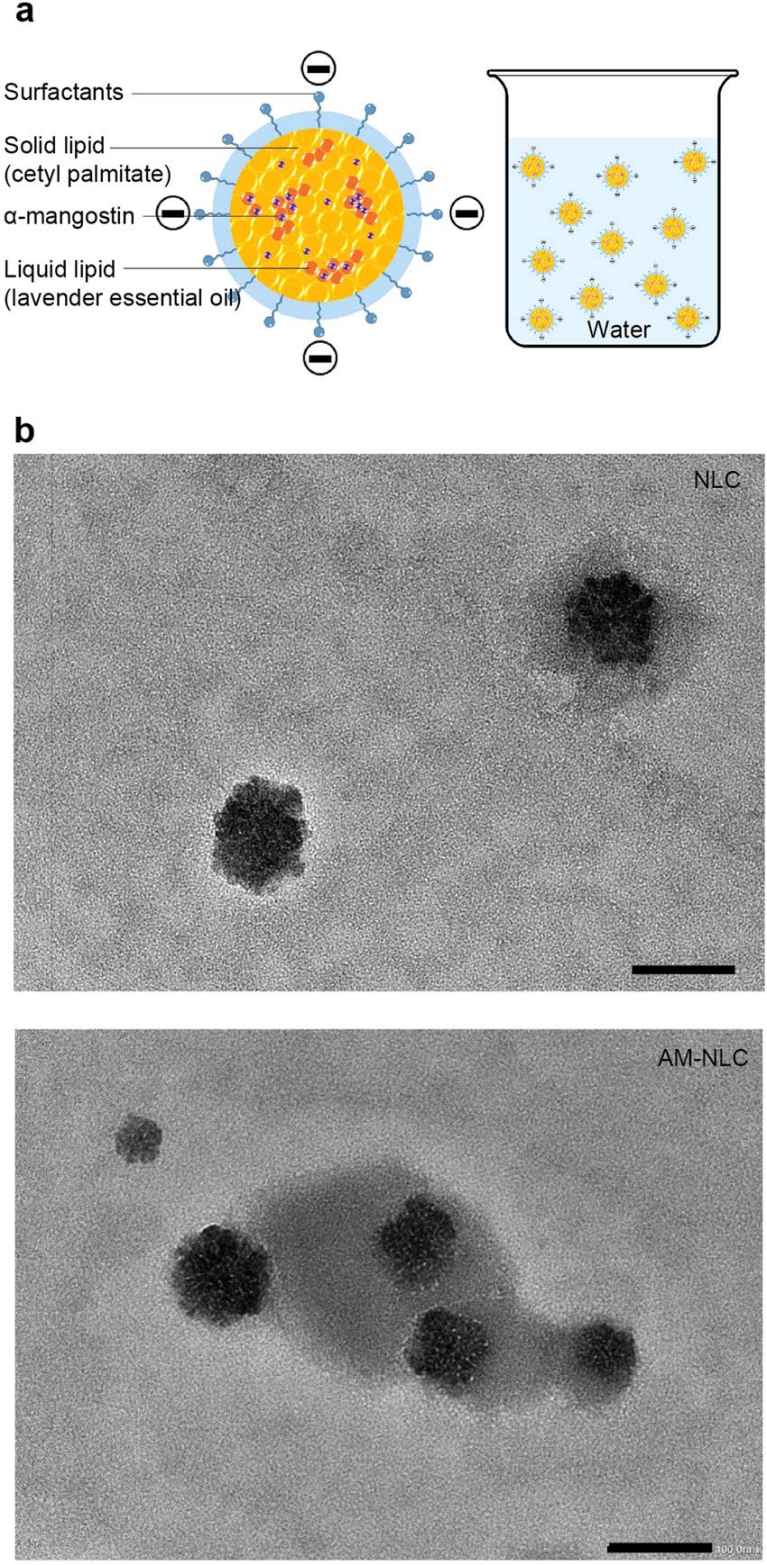
Physicochemical characteristics of α-mangostin-encapsulated NLC (AM-NLC) compared to plain NLC. (**a**) Schematic representation of AM-NLC. (**b**) Transmission Electron Micrograph of blank NLC and AM-NLC. (Scale bar: 100 nm).

A schematic diagram of the AM-NLC is shown in Fig. 1a. NLC and AM-NLC presented as a milky whitish and yellowish dispersion, respectively. The average encapsulation efficiency of AM was 82.60 ± 9.81%. The physicochemical properties of NLC and AM-NLC are shown in Table 1. Both formulations have similar size (approximately 100 nm), dispersity below 0.25, and negative zeta potential, regardless of the storage temperature and duration of storage used in this study. As shown in Fig. 1b, TEM image showed spherical particles of blank NLC and AM-NLC in the range of 80–100 nm which was in good agreement with the results from dynamic light scattering measurement.

**Table 1:** Physicochemical properties of NLC and AM-NLC after synthesis.

**Formulation**	**Duration of storage (day)**	**Storage Temperature (°C)**	**Average diameter (nm)**	**Dispersity**	**Zeta potential (mV)**
NLC	0	N.A.	98.52 ± 0.5027	0.184 ± 0.013	−37.5 ± 1.30
	60	25	100.00 ± 1.6920^ns^	0.204 ± 0.006^ns^	−39.4 ± 0.85^ns^
		45	97.68 ± 0.2921^ns^	0.186 ± 0.007^ns^	−37.6 ± 1.75^ns^
AM-NLC	0	N.A.	88.61 ± 0.9277	0.118 ± 0.017	−35.7 ± 2.51
	60	25	89.64 ± 1.2730^ns^	0.130 ± 0.016^ns^	−35.5 ± 1.45^ns^
		45	84.83 ± 0.3350**	0.101 ± 0.017^ns^	−25.4 ± 1.66***

Values were the means of three replicate samples. The data were presented as mean ± SEM. * were significant as compared to the day of production or day 0 (ns-not significant, **P < 0.01, ***P < 0.001).

### Kinetic release of AM-NLC via artificial membrane

**Figure 2:**
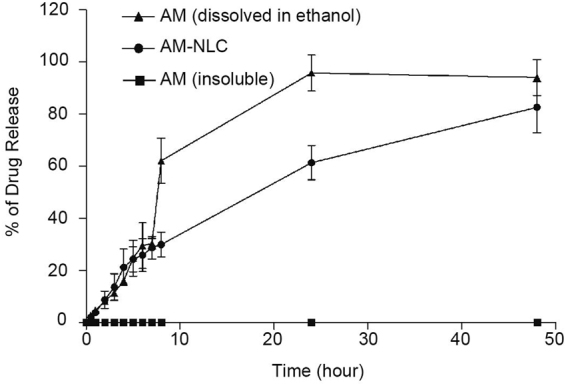
Cumulative percent AM release values over 50 hours, determined for the AM-NLC.

The cumulative release profiles of AM-NLC were obtained by quantifying the percentage of AM relative to the amount of AM originally loaded in the NLC. As shown in Fig. 2, the *in vitro* release of AM-NLC showed a biphasic drug release pattern for about 50 h. The release profile showed an initial release of about 25% AM during the 10 h, however, followed by sustained release for at least 50 h. On the contrary, the release profile of AM solution showed a prominent burst release of 90% drugs during the first 25 h (Fig. 2). The biphasic drug release is a characteristic for controlled drug delivery. The results also showed that poorly water-soluble AM could not diffuse pass through the membrane.

### *In vitro* cytotoxicity of the prepared NLC

**Figure 3:**
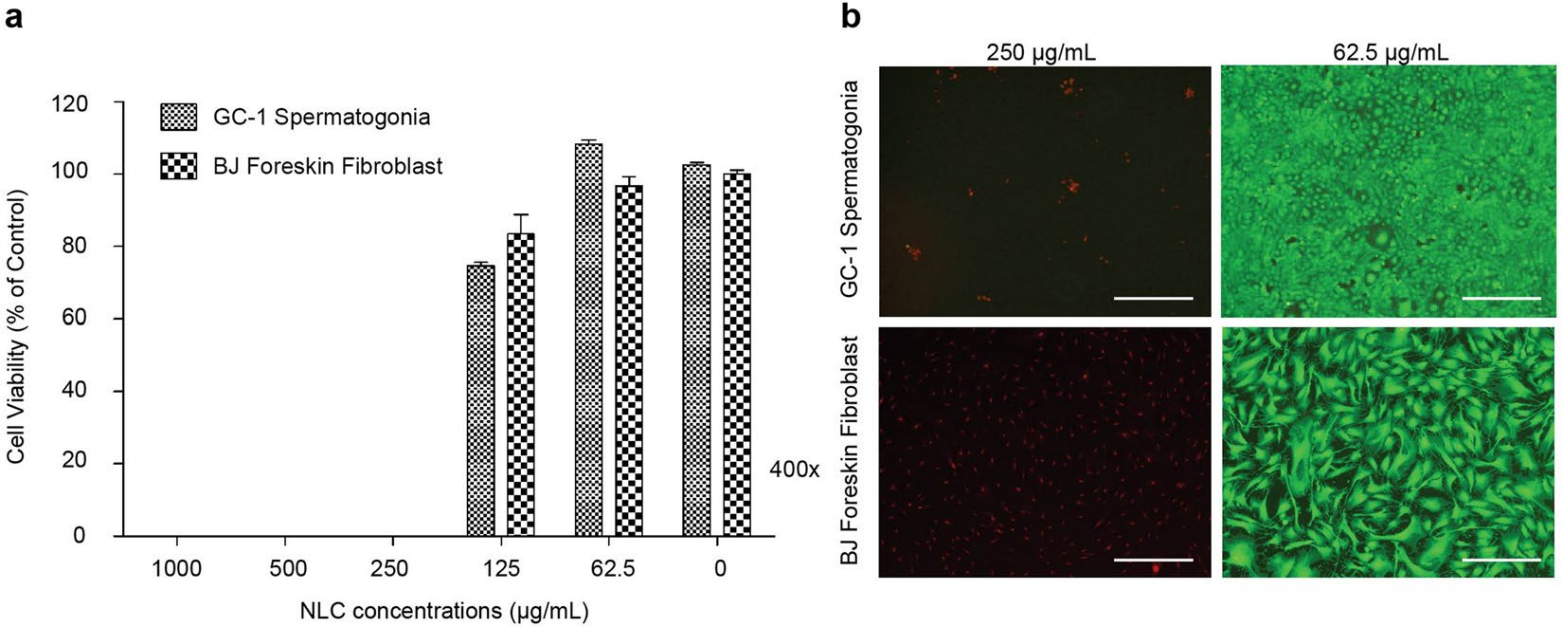
Cytotoxicity of blank NLC at various concentrations in GC-1 cells (mouse spermatogonium). (**a**) Following 24 h incubation with NLC, cell viability was determined by the CellTiter-Glo® cell viability assay. Mean cell viability was normalized to untreated controls, with the mean + SEM of n = 3, from one representative experiments of three independent experiments. Statistical analysis was performed using two way ANOVA, ***p < 0.001. (**b**) Morphological characteristics of mouse spermatogonium cell were visualized under the fluorescence microscope. Cells were stained with the reagents in the LIVE/DEAD® cell viability/cytotoxicity Assay Kit. Dead and live cells fluoresce red-orange and green, respectively. (Scale bar: 200 μm).

Before NLC could be used as a nanocarrier for AM delivery, the cytotoxicity of blank NLC was ascertained. The prepared NLC was composed of liquid and solid lipid, and surfactant(s). Therefore, we sought to determine the appropiate concentration of NLC for GC-1 spermatogonia cell line. The concentration of NLC was decreased by dilution of the NLC stock solution (100 mg/ml). Cell viability of GC-1 cells was examined after 24-h exposure to 0–1000 μg/mL NLC. As shown in Fig. 3a, the 250–1000 μg/mL NLC displayed less than 1% cell viability, increasing to 80% for 125 μg/mL, and no adverse effect (100% cell viability compared to the untreated cells) was observed for 62.5 μg/mL. Therefore, the optimal concentration for GC-1 cells was determined to be 62.5 μg/mL NLC. Based on this, further analysis was carried out by using this concentration. Similar results were observed in NLC-treated BJ Foreskin fibroblasts.

### Spermatogonium cell death induced by AM-NLC

**Figure 4:**
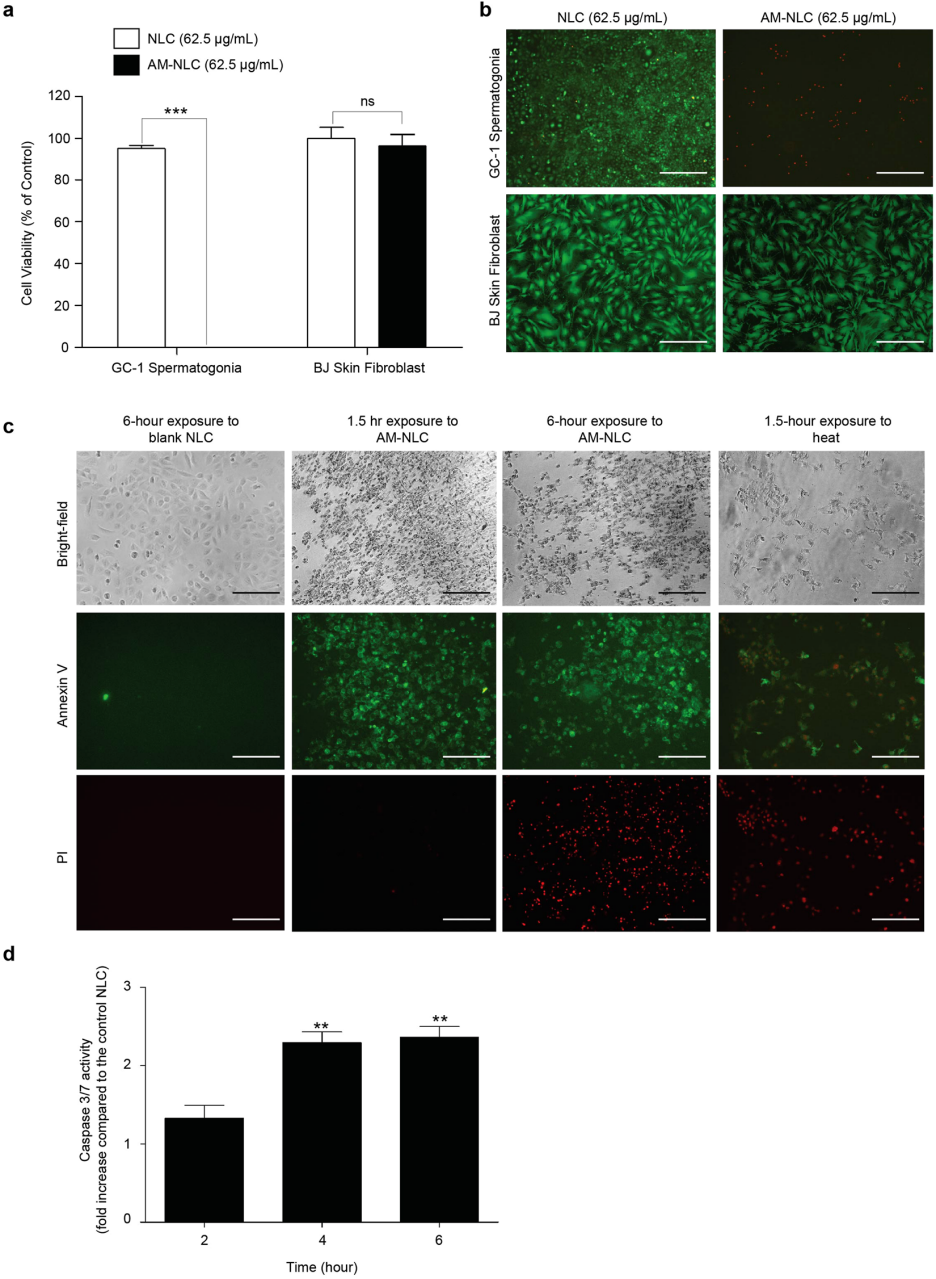
Male germ cell death induced by AM-NLC. (**a**) Cytotoxicity of AM-NLC or blank NLC in spermatogonia and foreskin fibroblast cell lines. Following 24 hr incubation with blank NLC or AM-NLC, cell viability was determined by the CellTiter-Glo® cell viability assay. Mean cell viability was normalised to non-treated controls. (**b**) Viability characteristics of mouse spermatogonium and foreskin fibroblast cells were visualized under the fluorescence microscope. Cells were stained with the reagents in the LIVE/DEAD® cell viability/cytotoxicity Assay Kit. (Scale bar: 200 μm). (**c**) Bright-field and fluorescence images display morphology and Annexin V/PI staining of spermatogenic cells after treatment with NLC or AM-NLC at diiferent time points and heat-induced necrotic cells for comparison. (Scale bar: 200 μm). (**d**) Determination of apoptosis. Caspases 3/7 activity were examined as indicators of apoptosis. Green and red fluorescence staining around cells indicates Annexin V-FITC and PI staining, respectively.

Next we investigated the effect of AM-NLC on proliferation of GC-1 spermatogonia cells and the mechanism. This was compared to blank NLC by using the predetermined concentration of NLC in order to show that the cytotoxicity of AM-NLC was due to the active compound, AM. Cells were treated with AM-NLC or blank NLC (62.5 μg/mL). This dilution was approximately equal to 6.25 μM of AM. Our results showed that the cell viability of AM-NLC-treated cells was drastically decreased as shown in Fig. 4a,b. In contrast, no cytotoxicity was observed in cells treated with blank NLC. Moreover, foreskin fibroblast cells were treated with the correspoding concentration of AM-NLC or blank NLC. No cytotoxicity was observed in foreskin fibroblast cells treated with blank NLC or AM-NLC, suggesting selective killing of spermatogonium cells induced by AM-NLC (Fig. 4a,b).

To gain a closer insight into AM-NLC-induced biological effects on GC-1 spermatogonia cells, Annexin V/PI staining and Caspases 3/7 activity were examined as indicators of apoptosis. To examine early and late apoptotic cells in AM-NLC-exposed cultures, fluorescence microscopy was performed using Annexin V-FITC and PI. As shown in Fig. 4c, control cultures treated with blank NLC remained unstained (Annexin V-FITC^−^/PI^−^). After 1.5 hour of exposure to AM-NLC, early apoptotic cells exhibited Annexin V-FITC^+^/PI^−^ staining patterns; whereas late apoptotic cells showed Annexin V-FITC^+^/PI^+^ staining patterns after 6 hour post-treatment. In contrast, after 1.5 hr induction of heat-triggered necrosis, cells exhibited strong PI staining, which identifies primary necrotic cells^19^. In addition, the results showed that AM-NLC significantly increased the activities of Caspases 3 and 7 (Fig. 4d).

### Cell death and survival in seminiferous tubules of cat testis after AM-NLC treatment

**Figure 5:**
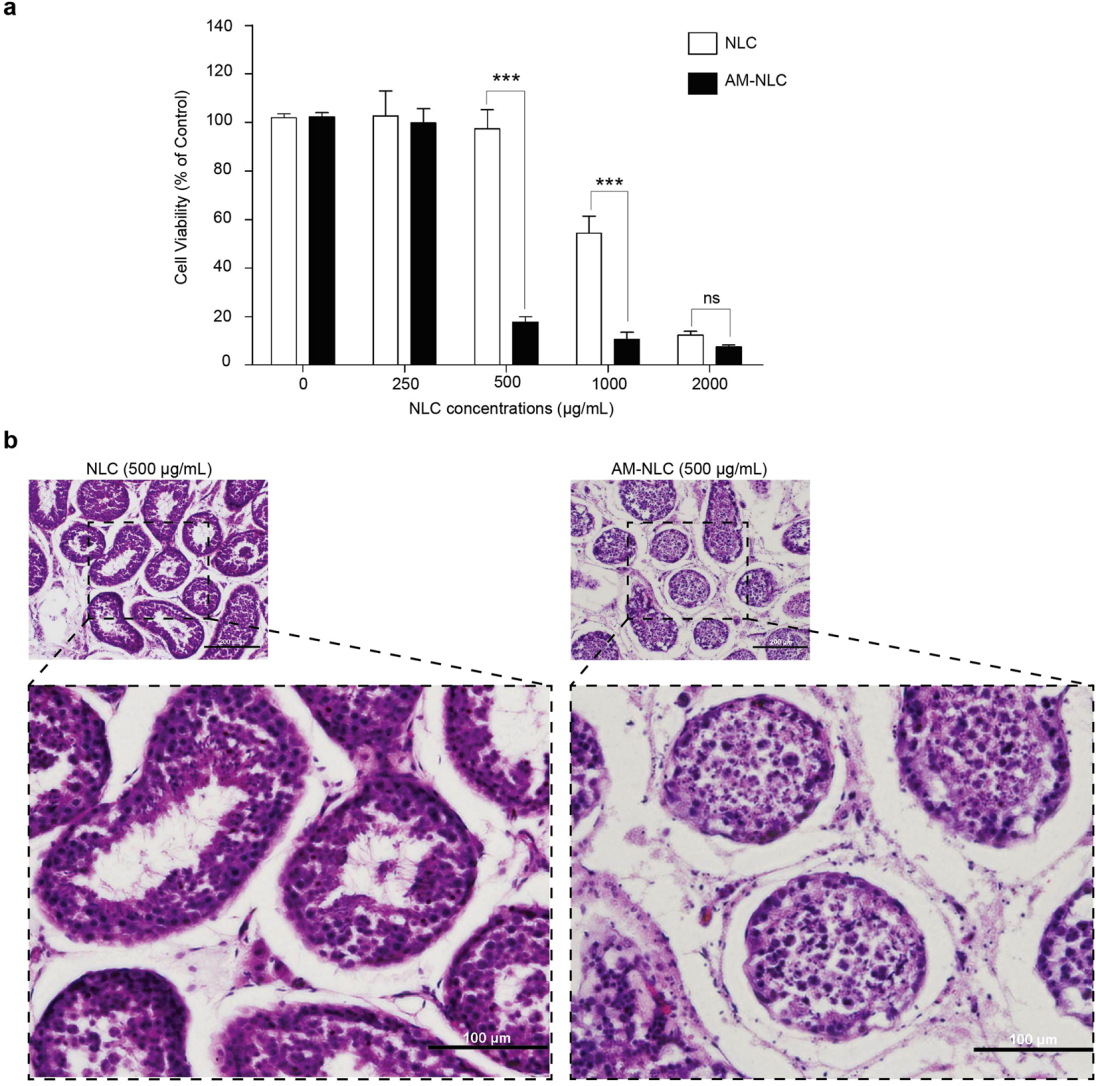
Cell death in seminiferous tubules of cat testis after AM-NLC treatment. (**a**) After 48 h incubation with various concentrations of blank NLC or AM-NLC, cell viability in seminiferous tubules was assessed by the CellTiter-Glo® cell viability assay. Mean cell viability was normalized to untreated controls, with the mean of n = 3 + SEM, from one representative experiments of three independent experiments. Statistical analysis was performed using two-way ANOVA, ***p < 0.001. (**b**) Photomicrograph of the seminiferous tubules. Testes tissue explants were obtained from the castrated testes. Images represent hematoxylin and eosin-stained sections of teticular explants after 48 h treatment with blank NLC or AM-NLC.

Treatment of intact seminiferous with AM-NLC was optimized to achieve high mortality by varying concentrations of NLC or AM-NLC (0–2,000 μg/mL). As shown in Fig. 5a, the result shows a significant cell death in seminiferous tubule’s testicle treated with 500 μg/mL AM-NLC. In addition, cell viability was decreased in a dose-dependent manner. The result also shows a difference in seminiferous tubules cross-section between the control blank NLC- and AM-NLC-treated groups. As shown in Fig. 5b, blank NLC-treated testicular explants showed mostly normal testicular anatomy with an orderly arrangement of germ cells and Sertoli cells and normal stages of spermatogenesis. In contrast, AM-NLC treatment induced the degeneration of germ cells within seminiferous tubules. Tubules were shrunken and greatly depleted of germ cells. **(**Fig. 5b).

### Effects of AM-NLC on NO and TNF-α Produced from LPS-stimulated RAW 264.7 Cells

**Figure 6:**
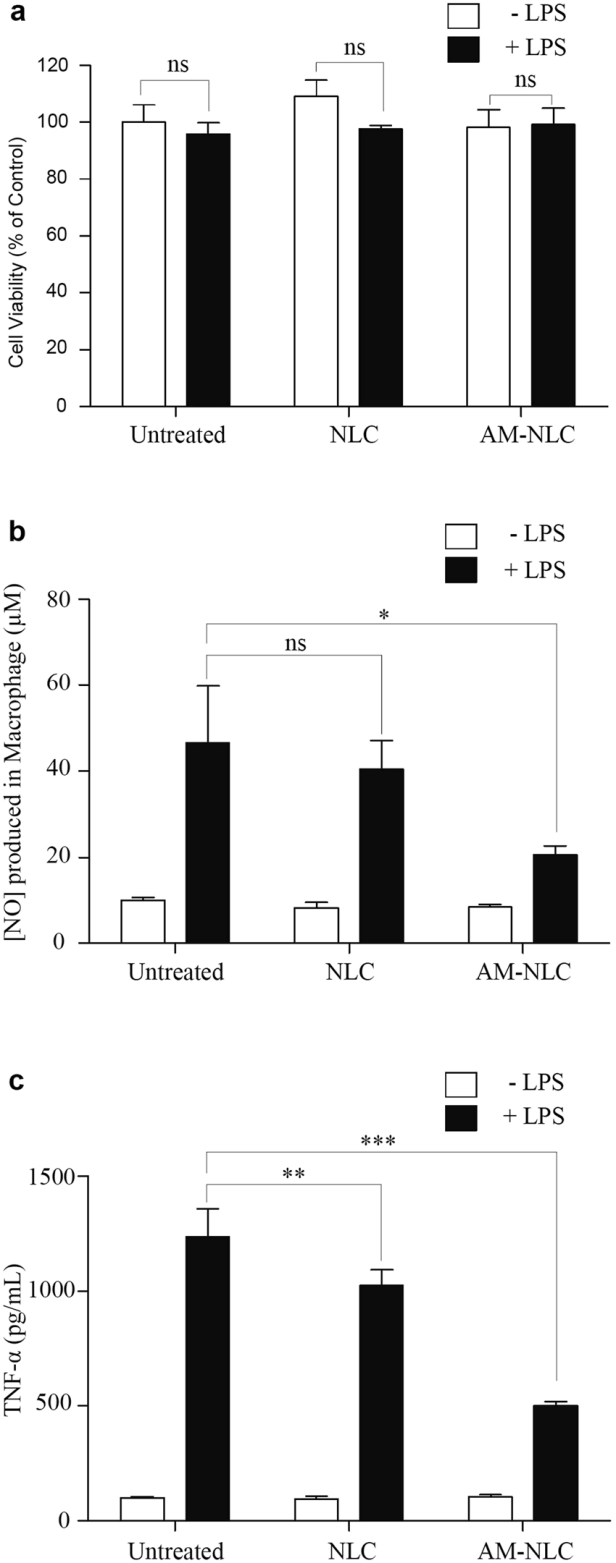
The effect of AM-NLC on the NO and TNF-αproduction in LPS-stimulated RAW 264.7 cells stimulated. Data are expressed as percentages of the control. Values represent mean + SEM, n = 3. Asterisks show significant differences as compared to control, p < 0.05 was considered as statistically significant as compared to control.

Raw264.7, a murine macrophage cell line has been extensively used for the screening of anti-inflammatory agents. In this study we investigated the effect of AM-NLC on nitric oxide (NO) and TNF-α production by LPS-stimulated macrophages. Our data demonstrated that AM-NLC (approved by cell viability test as non-toxic, as shown in Fig. 6a) caused a significant decrease in the accumulation of nitrites (Fig. 6b). In addition, TNF-α production by LPS-activated RAW 264.7 cells was measured in the presence of AM-NLC or blank NLC. In Fig. 6c, the data show that NLC containing essential lavender oil significantly reduced TNF-α production and that AM-NLC containing both AM and essential lavender oil have a stronger efficacy. We also confirmed the presence of active compounds by Gas Chromatography Mass Spectrometer (GCMS). The results of GCMS analyses of the essential oil are given in Supplementary Figure 1. The major constituents of the lavender oil were linalyl acetate and linallol. Taken together, the anti-inflammation property of AM-NLC is resulted from AM and essential lavender oil.

## Discussion

NLS and AM-NLS were successfully synthesized and investigated for their physiochemical properties (size, charge) and biological activities (apoptosis-induced male germ cell death and anti-inflammatory activity). The AM-NLC formulation obtained was found to have an EE value higher than 80%. Dispersity indicative of a monodisperse distribution (between 0.1 and 0.2), and a highly negative zeta potential (up to −30 mV), suggesting great stability in solution^20,21^. No significant changes were observed for 2 months after synthesis, when AM-NLC were stored at 25 °C. The high drug loading capacity and great stability is probably associated with the lipid composition of nanoparticles, since NLC present a disordered lipid matrix conferred by the presence of the liquid lipid^22^.

Another interesting finding was that the activity of AM-encapsulated NLC on GC-1 cell was greater than that of free AM. As shown in Supplementary Figure 2, the survival curve of spermatogenic cells after exposure to free AM showed that the amount of AM required to achieve 100% of cell death (LC100) was 60 μM. However, only 6.25 μM of AM in AM-NLC could induce 100% cell death of GC-1 cells, indicating that the NLC system enhanced the cytotoxicity of AM approximately 10 fold compared to free AM. Most importantly, the cytotoxicity of AM-NLC was due to the active compound (not from the NLC carrier) as previously determined by 100% cell viability when cells were treated with the corresponding amount of blank NLC. The enhanced activity of NLC associated AM may be related to the mode of entry of AM-NLC into the cells or a prolonged release of active compound from the lipid nanoparticles delivery systems^23^.

Recent developments in the field of animal contraception and sterilization have led to a renewed interest in non-surgical methods^24^. A previous study has reported the cytotoxicity of the commercially available zinc gluconate-based chemical sterilant that induces necrosis, recruits inflammatory cells, and triggers an immune response^2^. Moreover, necrotic process is potentially carcinogenic because it can stimulate cell proliferation and promote neoplastic progression^25^. It has been suggested that cell death mechanism would have to be apoptosis^2^.

Medicinal plants have been a major source from which s apoptosis-inducing agents are obtained^26^. Previous reports showed that bioactive compounds elicit apoptosis in cancer cells^27^. Therefore, many of these naturally occurring compounds have contributed to cancer treatment^28^. In this study, the application of apoptosis-inducing agents derived from herbal medicines have proven to be the effective antifertility compound that may have utility in non-surgical castration of male animals by intra-testicular injection.

Programmed cell death by apoptosis is more preferable than necrosis in eliminating unwanted cells because it does not trigger inflammation to neighboring cells^9^. In this regard, α-mangostin, a compound extracted from Mangosteen (*Garcinia mangostana* L) pericarp was used as a male germ cell apoptosis inducer. The present study elucidated the mechanism of apoptosis provoked by AM-NLC toward spermatogenic cells. According to the observations mentioned in this study, it can be concluded that AM-NLC has the ability to induce apoptosis in spermatogenic cells as confirmed by increased Caspases 3/7 activity as well as the patterns of Annexin V/PI staining for early apoptotic cells (Annexin V-FITC^+^/PI^−^) and late apoptotic cells (Annexin V-FITC^+^/PI^+^)^19^. This result seem to be in agreement with previous studies that α-mangostin is the potent apoptosis-inducing agent^29,30^.

Activation of macrophages triggers the production of NO and release of pro-inflammatory cytokines leading to inflammation^31^. Therefore, inhibition of NO and TNF-α production in LPS-stimulated macrophage cells is one of the possible ways to evaluate anti-inflammatory drugs^32^. Another aspect of AM-NLC, as presented in this study, is its potent anti-inflammatory activity. Our finding is consistent with a previous study showing that AM significantly inhibited nitric oxide (NO) production from LPS-stimulated RAW 264.7 cells^33^. Moreover, the use of lavender essential oil in combination with AM was found to have the synergistic effect as confirmed by the suppression of TNF-α production by NLC containing lavender essential oil and the stronger effect for the prepared AM-NLC containing both AM and lavender essential oil. We also confirmed the presence of these potent anti-inflammatory and anti-pyretic agents in lavender essential oil as confirmed by GCMS (Supplementary Figure 1). This results are consistent with other research which found the anti-inflammatory, analgesic, anti-fungal, anti-bacterial and anti-microbial agents in lavender essential oil^34^. From this data it can be suggested that the prepared AM-NLC may be useful in preventing inflammation mediated by excessive production of NO and TNF-α.

The nanovation (innovation + nanotechnology), as presented here, is an improved version of antifertility compound for non-surgical castration of male animals. Specifically, we reported in this study the preparation of α-mangostin-loading NLC as well as their physicochemical and biological properties. More importantly, this study demonstrated the feasibility of AM-NLC by using *in vitro* and *ex vivo* model. Despite these promising results, a large range of related clinical parameters needed to be measured and monitored over a period of time. Further research should be undertaken to investigate the *in vivo* animal studies in order to determine the effectiveness of AM-NLC for chemical castration by intratesticular injection. In addition to related clinical significances, such as changes in the testis, testosterone levels, reduces sperm motility and male fertility, adverse side-effects (pain and inflammation) and long-term safety and should be evaluated in parallel.

## Conclusions

The innovative strategy presented here is a new version of nanomedicine-based sterilant for non-surgical castration of male animals by using α-mangostin which simultaneously serves as a male germ cell apoptosis inducer and an anti-inflammatory agent). In the cell model, AM-NLC reduced the levels of pro-inflammatory mediators including NO and TNF-α induced by LPS. In addition, this study found apparent advantage of using lavender essential oil to improve the anti-inflammation of the prepared AM-NLC. This nanomedicine-based sterilant would be a promising platform that may have utility in non-surgical castration of male animals by intra-testicular injection.

## Electronic supplementary material

Supplementary Information
