# Supplementary material for: Nanocarrier-mediated delivery of α-mangostin for non-surgical castration of male animals
Source: Sci Rep. 2017 Nov 24;7:16234. doi: 10.1038/s41598-017-16563-3 (PMC5701201; doi:10.1038/s41598-017-16563-3)
Supplement: Supplementary file 1 — Supplementary Information [file 41598_2017_16563_MOESM1_ESM.pdf]

Supplementary Information

Title

Nanocarrier-mediated delivery of alpha-mangostin for non-surgical castration of male animals

Authors

Jakarwan Yostawonkul[1], Suvimol Surassmo[1], Katawut Namdee[1], Mattaka Khongkow[1], Chatwalee Boonthum[2], Sasithon Pagseesing[2], Nattika Saengkrit[1], Uracha Ruktanonchai[1], Kaywalee Chatdarong[2], Suppawiwat Ponglowhapan[2], and Teerapong Yata[1]*

Authors' affiliation

[1]National Nanotechnology Centre (NANOTEC), National Science and Technology Development Agency, Pathumthani, Thailand

[2]Department of Obstetrics, Gynaecology and Reproduction, Faculty of Veterinary Science, Chulalongkorn University, Bangkok, Thailand

Correspondence:

Teerapong Yata

National Nanotechnology Centre (NANOTEC), National Science and Technology Development Agency, 111 Thailand Science Park, Paholyothin Rd., Klong Luang, Pathumthani 12120, Thailand.

Phone +66 2117 6549, Fax +66 2564 6985

E-Mail: teerapong@nanotec.or.th

Materials

Cetyl palmetate was purchased from Sabowax S.p.A. (Levate BG, Italy). Miglyol 812 was purchased from Sasol Germany GmbH. (Hamburg, Germany). Montanov 82 was purchased from Adiniop Co. Ltd. (Bangkok, Thailand). Polyoxyethylene (20) sorbitan monolaurate was purchased from PC intertrade. (Bangkok, Thailand). Lavender oil were purchased from thai-china flavours and fragrances industry Co. Ltd. (Bangkok, Thailand). Poloxamer 188 was purchased from Croda Co. Ltd. (Bangkok, Thailand). Glycerol was purchased from Chem plus trading, (Bangkok, Thailand). α-mangostin was purchased from Honsea sunshine Bio science and technology Co. Ltd. (Guangzhou, China). All other chemicals and reagents used in these experiments were of analytical grade. The murine spermatogonia (GC-1), and the murine macrophage RAW264.7 cell lines were obtained from American Type Culture Collection (ATCC). Testicular explants were obtained from castrated testes of cats. Lipopolysaccharide (LPS) were purchased from Sigma. The Dulbecco's modified eagle medium (DMEM), Roswell Park Memorial Institute (*RPMI*) *1640 medium,* antibiotics, and fetal bovine serum (FBS) were purchased from Gibco. LIVE/DEAD® Viability/Cytotoxicity kit was obtained from Invitrogen. CellTiter-Glo® Luminescent Cell Viability and Caspase-Glo® 3/7 assay system were provided by Promega.

Gas chromatography and mass spectrometry analysis

Essential oils were analyzed by gas chromatography coupled with mass spectrometer (GC-MS). GC–MS analyses were carried out using Agilent Technology gas chromatograph (7890 Gas chromatograph, California, USA) equipped with a capillary column DB-5 (Agilent J&W, Agilent technologies Inc., California, USA) with 30 m × 0.25 mm i.d. × 0.25 mm film thickness coated with (5%-Phenyl)-methylpolysiloxane. Helium gas was used as the carrier at a flow rate of 1 mL/min. The oven temperature program was 60°C for 2 min then 5˚C/min to 220˚C with 3 minutes of holding time, and finally to 280˚C at a heating rate of 30˚C/min. The 1 µL of samples was injected. Injector and detector temperatures were 250 and 2 80˚C, respectively. Mass spectrometry (MS5975 mass spectrometer, California, USA) were used as detector. The chemical structures were identified by comparing mass data with standard library data (Willey & Sons, Inc., Hoboken, NJ)

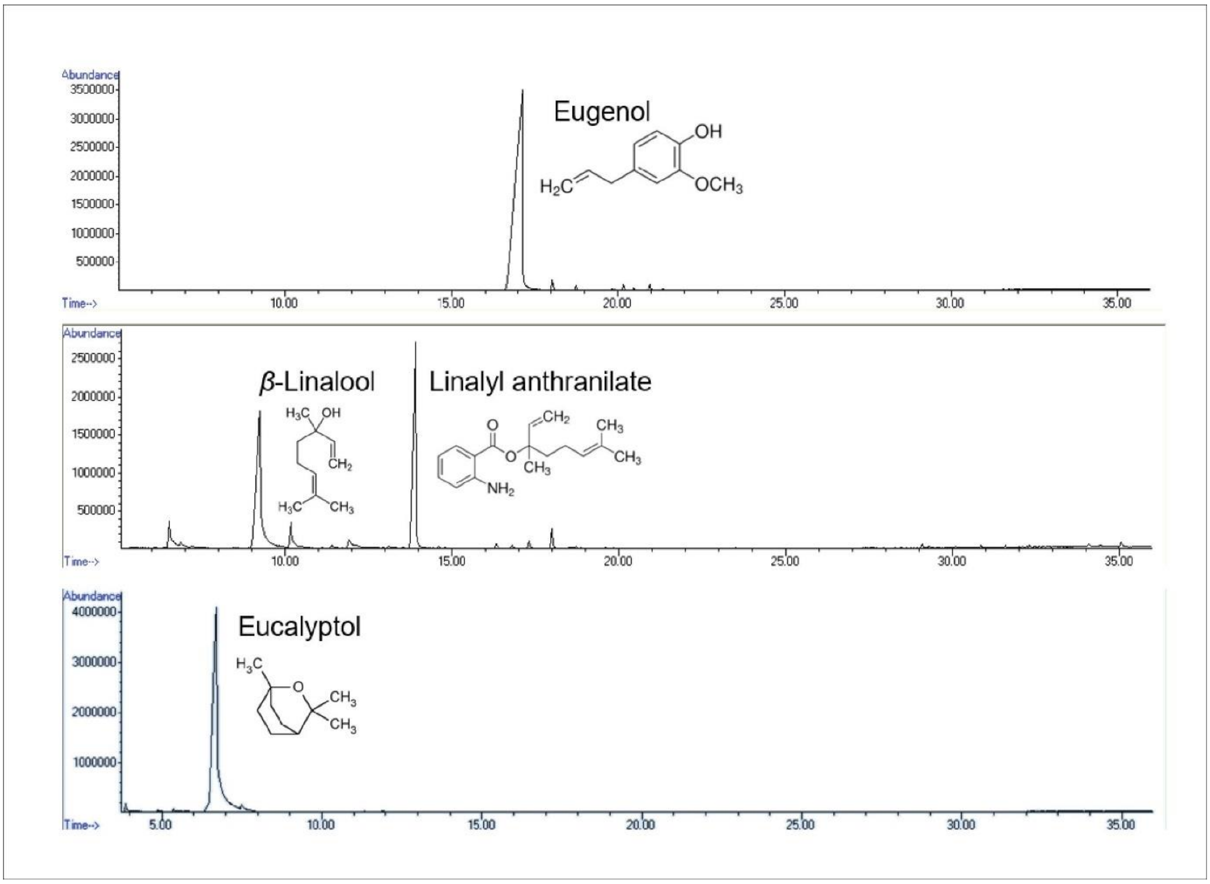

Supplementary Figure 1. Chromatogram generated by a gas chromatography.

(**a**) Clove oil (**b**) lavender oil (**c**) eucalyptus oil.

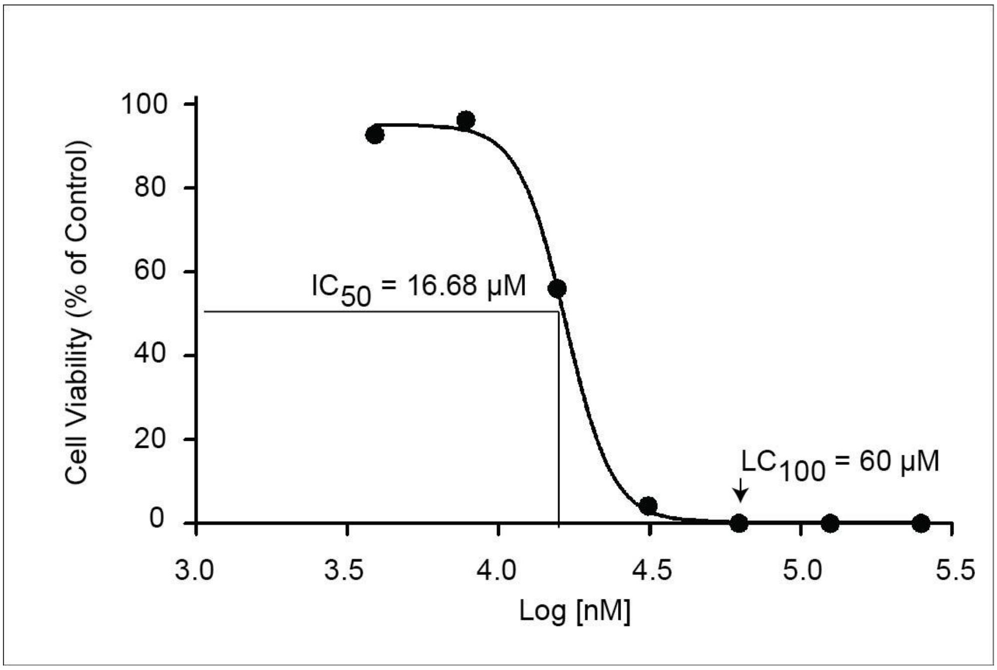

Supplementary Figure 2. Determination of the absolute lethal concentration (LC_{100}) value (in μM) for α-mangostin against spermatogonia cells. Spermatogonia cell viability was determined after 24 hr exposure to various concentrations of α-mangostin. The IC_{50} and LC_{100} values are defined as the concentration that causes 50 and 100% growth inhibition in treated cells when compared to the control untreated cells, respectively.
